# Postoperative Takotsubo Cardiomyopathy in a Young Woman: A Case Report

**DOI:** 10.1002/ccr3.73339

**Published:** 2026-08-23

**Authors:** Nadia Siddiq, Zoreiz Zahid Cheema, Sana Samad Khan, Aruba Arshad, Abdul Rasheed Awan, Saeedullah Shah, Muhammad Usman

**Affiliations:** ^1^ Shifa Clinical Research Centre Shifa International Hospital Islamabad Pakistan; ^2^ Department of Medicine Allama Iqbal Medical College/Jinnah Hospital Lahore Pakistan; ^3^ Shifa College of Medicine Islamabad Pakistan; ^4^ Department of Cardiology Shifa International Hospital Islamabad Pakistan; ^5^ Department of Medicine, Division of Hospital Medicine University of Wisconsin Hospital & Clinics Madison Wisconsin USA

**Keywords:** perioperative cardiac dysfunction, postoperative complication, small‐bowel obstruction, stress‐induced cardiomyopathy, Takotsubo cardiomyopathy, transfusion reaction

## Abstract

Takotsubo cardiomyopathy (TCM) can occur in young adults exposed to extreme physical stress, such as surgery, sepsis, and transfusion reactions. Mechanical recovery may precede electrical normalization, with ECG abnormalities persisting despite improved left ventricular ejection fraction (LVEF). Early recognition and monitoring are crucial to prevent misdiagnosis and guide management.

## Graphical abstract

Timeline of the patient's clinical course.

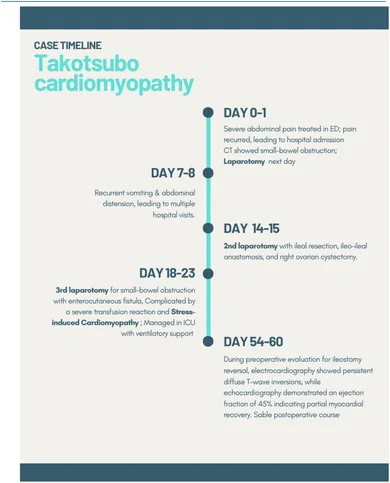

## 1 Introduction

Takotsubo cardiomyopathy (TCM), also known as “broken heart syndrome” or stress‐induced cardiomyopathy, is typically considered a diagnosis of exclusion. It accounts for approximately 1%–2% of patients initially suspected of having acute coronary syndrome. Takotsubo syndrome predominantly affects postmenopausal women; however, younger individuals may also be affected. In a multicenter International Takotsubo Registry analysis of 2098 patients, only 11.5% of cases occurred in patients ≤ 50 years of age [1, 2]. The Mayo Clinic diagnostic criteria, widely used for confirming stress cardiomyopathy, require transient left ventricular wall motion abnormalities (often beyond a single vascular territory and sometimes following stress), absence of obstructive coronary disease or plaque rupture, new ECG changes or mild troponin elevation, and exclusion of pheochromocytoma and myocarditis [3]. Clinically, patients with TCM often demonstrate characteristic ECG abnormalities. ST‐segment elevation and T‐wave inversion are the most common findings, while ST‐segment depression is less frequent [4]. We report a rare case of stress‐induced cardiomyopathy in a young woman following multiple abdominal surgeries complicated by sepsis and a transfusion reaction. This unique combination of intense physical stressors precipitated severe myocardial dysfunction. Intriguingly, electrocardiographic changes persisted long after echocardiographic recovery, revealing a rare and critical dissociation between electrical and mechanical myocardial healing processes. This case not only deviates from established demographic patterns but also highlights the unpredictable and complex etiology of stress cardiomyopathy.

## 2 Case Presentation

**FIGURE 1:**
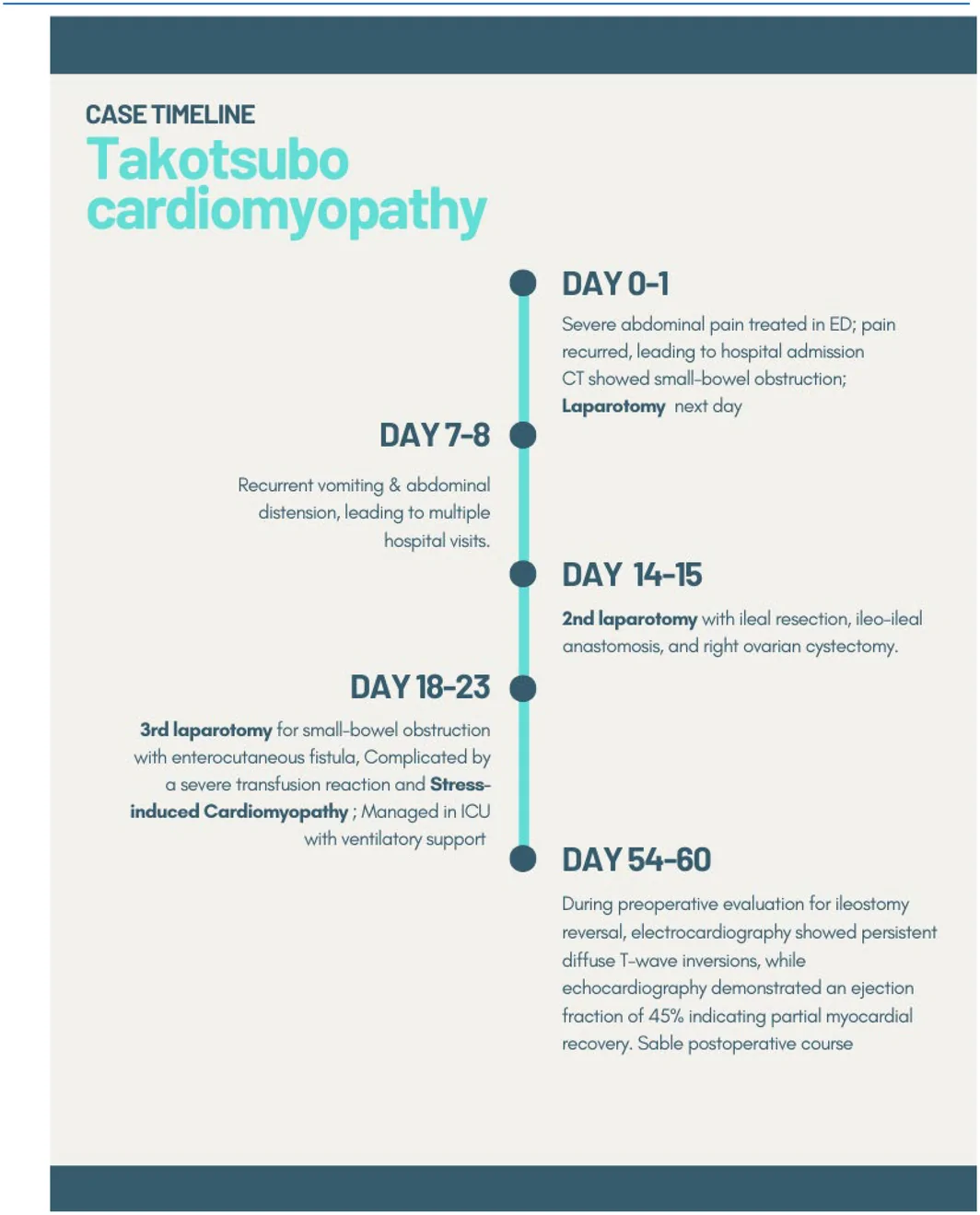
Timeline summarizing the clinical course of postoperative Takotsubo cardiomyopathy following multiple abdominal surgeries.

**FIGURE 2:**
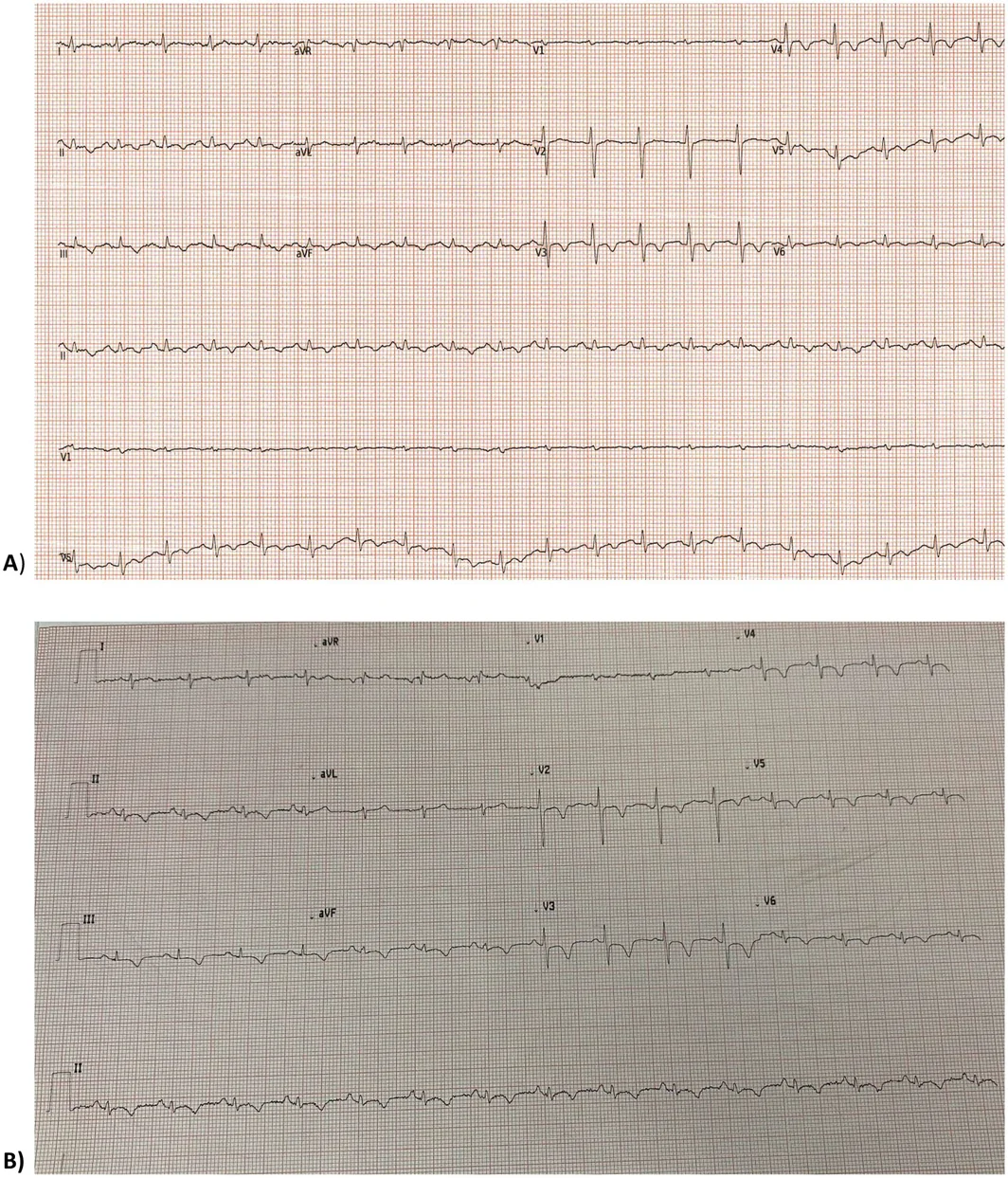
(A) Patient's electrocardiogram shows sinus rhythm with rightward axis deviation and widespread T‐wave flattening and inversion, most prominent in the anterolateral (V2–V6) and inferior (II, III, aVF) leads. (B) Patient's electrocardiogram shows sinus tachycardia with persistent T‐wave inversion in the anterolateral (V3–V6) and inferior (II, III, aVF) leads.

A woman in her mid‐20s, previously healthy, presented to the emergency department with sudden‐onset, severe, generalized abdominal pain. This presentation, together with the abdominal imaging, is taken as Day 1 of her clinical course. She was managed symptomatically and discharged following transient improvement. However, her pain recurred later the same evening, prompting re‐evaluation at the same facility. A contrast‐enhanced CT of the abdomen demonstrated small‐bowel obstruction, and on Day 2 she subsequently underwent an emergency exploratory laparotomy the next day. The overall chronology of the patient's clinical course is summarized in Figure 1. Although she initially recovered uneventfully, by Day 7–8 she developed recurrent vomiting and progressive abdominal distension. Over the subsequent days, she sought care at multiple hospitals due to persistent and worsening symptoms. On Day 14–15, persistent abdominal obstruction warranted a second (re‐look) laparotomy, which included ileal resection with primary ileo‐ileal anastomosis and right ovarian cystectomy. Although she was initially stable in the immediate postoperative period, within days she developed persistent vomiting, progressive abdominal distension, and purulent discharge from the surgical wound, which indicated breakdown of the anastomosis with intra‐abdominal sepsis. On Day 18, she presented to our tertiary‐care hospital with severe abdominal pain, purulent wound discharge, and marked abdominal distension. CT imaging showed small‐bowel obstruction with an enterocutaneous fistula. She underwent an exploratory laparotomy with peritoneal washout and formation of a double‐barrel ileostomy under general anesthesia. Intraoperative findings confirmed purulent and feculent peritonitis that involved all four quadrants, a blown‐out ileal anastomosis, and a 0.5 cm proximal ileal perforation. These discoveries accounted for the recurrent obstructive symptoms and progressive sepsis that had warranted each repeat laparotomy. During surgery, a severe transfusion reaction occurred, requiring immediate cessation of transfusion and institution of inotropic and ventilatory support. Microbiological culture of the surgical‐site discharge yielded extensively drug‐resistant (XDR) *Escherichia coli* and *Candida tropicalis* . The *E. coli* isolate was susceptible only to minocycline, tigecycline, and colistin, while resistant to all tested cephalosporins, carbapenems, and fluoroquinolones. Strict infection‐control precautions were implemented, and targeted antimicrobial therapy was guided by susceptibility results. Postoperatively, she developed haemodynamic instability with markedly elevated cardiac troponin levels, prompting a provisional diagnosis of acute myocardial infarction. Echocardiography subsequently demonstrated findings consistent with stress‐induced (Takotsubo) cardiomyopathy, including reduced ejection fraction (35%) with apical and septal wall‐motion abnormalities and basal sparing. A small pericardial effusion was also noted. A small (8 mm) insignificant pericardial effusion was also observed. Although sepsis and the intraoperative transfusion reaction may have contributed to physiological stress burden, they were considered precipitating factors rather than alternative explanations for the observed echocardiographic pattern. She required intensive‐care and ventilatory support for 5 days. Once clinically stable, infection‐control measures were reinforced, and targeted antimicrobial therapy was initiated based on culture sensitivities (minocycline, tigecycline, and colistin). During pre‐operative cardiac optimisation prior to ileostomy reversal, guideline‐directed medical therapy for Takotsubo cardiomyopathy was initiated, consisting of nebivolol 2.5 mg, losartan 25 mg, and eplerenone 50 mg once daily, each started at half the usual dose to minimize hypotensive risk. In view of the reduced ejection fraction and heart failure state, loop diuretic therapy was also administered as clinically indicated. Three weeks after the third surgery, during readmission for acute kidney injury and high‐output ileostomy, a 12‐lead ECG (Figure 2A) showed sinus rhythm with rightward axis deviation and widespread T‐wave flattening and inversion, most prominent in the anterolateral (V2–V6) and inferior (II, III, aVF) leads. These findings were consistent with the residual electrical signature of Takotsubo cardiomyopathy. Laboratory investigations revealed evidence of hepatic stress, anemia, and mild leukocytosis, consistent with ongoing sepsis and systemic inflammatory response. Detailed hematological, liver‐function, renal‐function, and electrolyte profiles are summarized in Tables 1, 2, 3. By approximately Day 55, during pre‐operative cardiac evaluation prior to ileostomy reversal, a repeat ECG demonstrated sinus tachycardia with persistent T‐wave inversion in the anterolateral (V3–V6) and inferior (II, III, aVF) leads (Figure 2B). Follow‐up echocardiography demonstrated improvement in left‐ventricular systolic function, with an ejection fraction of approximately 45% and resolution of the previously noted pericardial effusion. These findings indicated ongoing but improving myocardial repolarisation abnormalities, consistent with the natural recovery phase of stress‐induced cardiomyopathy.

## 3 Investigations

**TABLE 1:** Complete blood count (CBC).

Parameter	Result	Ref. range
WBC	5890/μL	4000–10,000
RBC	3.71 m/μL	3.8–4.8
Hemoglobin	10.9 g/dL	12–15
HCT	32.2%	36%–46%
MCV	86.8 fL	83.0–101.0 fL
MCH	29.4 pg	27–32 pg
MCHC	33.9 g/dL	31.5–34.5 g/dL
Platelets	221,000/μL	150–450 × 10^3^
Neutrophils	90%	40%–80%
Lymphocytes	5%	20%–40%
Monocytes	5%	2%–10%
Eosinophils	0%	1%–6%
Basophils	0%	< 1.0%

**TABLE 2:** Liver function test (LFTs).

Parameter	Result	Ref. range
AST (SGOT)	58 U/L	F ≤ 35
ALT (SGPT)	47 U/L	F ≤ 35
Alkaline Phosphatase	62 U/L	40–130
Total Bilirubin	3.85 mg/dL	≤ 1.2
Direct Bilirubin	2.24 mg/dL	≤ 0.30
Gamma GT	48 U/L	F ≤ 40

**TABLE 3:** Electrolyte and renal function tests (RFTs).

Parameter	Result	Ref. range
Sodium	142 mEq/L	136–145
Potassium	4.2 mEq/L	3.5–5.1
Chloride	108 mEq/L	98–107
Bicarbonate (HCO_3_)	18 mEq/L	22–29
BUN	6 mg/dL	6–20
Urea	12.84 mg/dL	16.6–48.5
Creatinine	0.44 mg/dL	F 0.57–1.11
eGFR	128.55 mL/min/1.73 m^2^	> 60

## 4 Treatment

The patient was managed in the intensive‐care unit for 5 days following the third surgery, receiving ventilatory and inotropic support. Once clinically stable, infection‐control measures were reinforced, and targeted antimicrobial therapy was initiated based on culture sensitivities (minocycline, tigecycline, and colistin). During pre‐operative cardiac optimisation prior to ileostomy reversal, guideline‐directed medical therapy for Takotsubo cardiomyopathy was initiated, consisting of nebivolol 2.5 mg, losartan 25 mg, and eplerenone 50 mg once daily, each started at half the usual dose to minimize hypotensive risk.

## 5 Outcome and Follow‐Up

Following ileostomy reversal, the patient's postoperative course was uneventful, with gradual resumption of oral intake and improvement in nutritional status. However, she was briefly readmitted with dehydration and anemia secondary to a high‐output stoma, which improved following fluid resuscitation and blood transfusion. At cardiology review on the same day as the recovery echocardiogram (approximately Day 55), by which time the LVEF had improved to approximately 45% and the diagnosis was documented as Takotsubo cardiomyopathy with significant recovery, guideline‐directed medical therapy was continued at the reduced doses described above: nebivolol at 2.5 mg, losartan at 25 mg, and eplerenone 50 mg, each at half‐dose daily for 30 days, with a plan for subsequent dose reduction and a repeat echocardiogram. She was discharged in stable condition on this regiment together with short‐course oral antibiotics and supportive therapy.

## 6 Discussion

This case underscores the multifactorial nature of stress cardiomyopathy, where overlapping physical insults can act synergistically to provoke transient myocardial dysfunction [5]. In our patient, the combined effects of repeated abdominal surgeries, sepsis, and a transfusion reaction produced a profound neurohormonal stress response culminating in catecholamine‐mediated myocardial stunning. This pattern highlights that even in the absence of emotional stress, Takotsubo cardiomyopathy (TCM) can occur in younger patients exposed to extreme physiological stress [6].

The pathophysiology of TCM is increasingly recognized as a neurocardiogenic syndrome arising from catecholamine‐induced myocardial injury. Acute physical or emotional stress triggers intense sympathetic discharge, leading to transient myocardial stunning through β‐adrenergic overstimulation, cyclic AMP–mediated calcium overload, and oxidative stress [7]. These mechanisms produce reversible myocardial dysfunction despite unobstructed coronary arteries. Microvascular dysfunction and endothelial injury may further contribute to localized hypoperfusion and regional wall‐motion abnormalities [8]. In addition to mechanical dysfunction, electrophysiological remodeling may manifest as QT‐interval prolongation and deep T‐wave inversions.

Mechanical recovery in TCM typically occurs within days to weeks, whereas electrical recovery often follows a slower trajectory as repolarization and autonomic tone normalize. Cardiac MRI studies have demonstrated persistent myocardial edema and subtle delayed enhancement even after normalization of left ventricular function, suggesting residual subclinical injury [9]. This mechanical–electrical dissociation explains the persistence of T‐wave inversions and QT prolongation beyond echocardiographic recovery [10]. In our patient, persistent ECG abnormalities despite normalized ventricular function illustrate this phenomenon and emphasize the importance of continued electrocardiographic monitoring during recovery.

Perioperative and transfusion‐related stress are increasingly recognized as important precipitants of TCM. Surgical interventions and anesthesia provoke intense sympathetic activation through factors like pain, hemodynamic instability, and neurohormonal surges. In complex surgical settings, transfusion reactions and immune‐mediated inflammatory events can further contribute to this stress, precipitating TCM development [11]. The temporal pattern of perioperative TCM is distinctive: approximately 30%–40% of cases occur intraoperatively during anesthesia or surgery, with the majority presenting during emergence or within the first postoperative day. This clustering of events predominantly within the immediate perioperative window, from induction to the first 24 h postoperatively, underscores the critical need for heightened cardiac surveillance throughout intraoperative and early recovery periods [12].

In our patient, the development of TCM following multiple abdominal surgeries complicated by sepsis and a transfusion reaction likely reflects the complex physiological stress response described above. Recognition of such multifactorial triggers is essential, particularly in perioperative and critically ill patients presenting with ECG changes or hemodynamic instability despite unobstructed coronary arteries. Maintaining a high index of suspicion can prevent misdiagnosis and reduce unnecessary invasive interventions. Our findings reinforce that TCM may occur outside the classic demographic profile, underscoring the need for vigilance in diverse clinical settings. Ongoing ECG and imaging follow‐up are advisable to ensure complete functional recovery and detect delayed electrical normalization. Awareness of this variability is essential for accurate diagnosis and optimal supportive management.

## 7 Conclusion

This case illustrates that Takotsubo cardiomyopathy can occur even in young women when exposed to extreme physiological stress. Multiple concurrent insults, including surgery, sepsis, and a transfusion reaction, may act synergistically to precipitate stress‐induced cardiomyopathy in patients outside the typical demographic profile. Accurate differentiation from acute coronary syndrome or septic cardiomyopathy requires recognition of the characteristic echocardiographic pattern and careful integration of clinical context. Importantly, mechanical improvement in left‐ventricular function may precede full electrical recovery, underscoring the need for continued ECG surveillance despite apparent clinical stabilization. Early recognition through multidisciplinary collaboration can prevent unnecessary invasive interventions and ensure optimal supportive management in perioperative cardiac dysfunction.

## Author Contributions

**Nadia Siddiq:** conceptualization, data curation, investigation, project administration, validation, visualization, writing – original draft, writing – review and editing. **Zoreiz Zahid Cheema:** conceptualization, data curation, visualization, writing – original draft, writing – review and editing. **Sana Samad Khan:** data curation, writing – original draft, writing – review and editing. **Aruba Arshad:** data curation, writing – original draft, writing – review and editing. **Abdul Rasheed Awan:** data curation, writing – original draft, writing – review and editing. **Saeedullah Shah:** conceptualization, data curation, investigation, project administration, supervision, validation, visualization, writing – review and editing. **Muhammad Usman:** supervision, validation, visualization, writing – review and editing.

## Funding

The authors have nothing to report.

## Consent

Written informed consent was obtained from the patient for publication of this case report and accompanying images.

## Conflicts of Interest

The authors declare no conflicts of interest.

## Data Availability

The data that support the findings of this case report are available from the corresponding author upon reasonable request. The data are not publicly available due to patient privacy and ethical restrictions associated with clinical case reporting.

## References

[1] C. Templin , J. R. Ghadri , J. Diekmann , et al., “Clinical Features and Outcomes of Takotsubo (Stress) Cardiomyopathy,” New England Journal of Medicine 373, no. 10 (2015): 929–938. 26332547 10.1056/NEJMoa1406761

[2] K. A. Bybee , T. Kara , A. Prasad , et al., “Systematic Review: Transient Left Ventricular Apical Ballooning: A Syndrome That Mimics ST‐Segment Elevation Myocardial Infarction,” Annals of Internal Medicine 141, no. 11 (2004): 858–865. 15583228 10.7326/0003-4819-141-11-200412070-00010

[3] I. Martín de Miguel , I. J. Núñez‐Gil , A. Pérez‐Castellanos , et al., “Electrocardiographic Characteristics and Associated Outcomes in Patients With Takotsubo Syndrome. Insights From the RETAKO Registry,” Current Problems in Cardiology 46, no. 8 (2021): 100841. 33994036 10.1016/j.cpcardiol.2021.100841

[4] J. R. Ghadri , I. S. Wittstein , A. Prasad , et al., “International Expert Consensus Document on Takotsubo Syndrome (Part I): Clinical Characteristics, Diagnostic Criteria, and Pathophysiology,” European Heart Journal 39, no. 22 (2018): 2032–2046. 29850871 10.1093/eurheartj/ehy076 PMC5991216

[5] S. Baltzer Nielsen , S. Stanislaus , K. Saunamäki , C. Grøndahl , J. Banner , and M. B. Jørgensen , “Can Acute Stress Be Fatal? A Systematic Cross‐Disciplinary Review,” Stress 22, no. 3 (2019): 286–294. 30767612 10.1080/10253890.2018.1561847

[6] L. Zhang and I. L. Piña , “Stress‐Induced Cardiomyopathy,” Heart Failure Clinics 15, no. 1 (2019): 41–53. 30449379 10.1016/j.hfc.2018.08.005

[7] F. Pelliccia , J. C. Kaski , F. Crea , and P. G. Camici , “Pathophysiology of Takotsubo Syndrome,” Circulation 135, no. 24 (2017): 2426–2441. 28606950 10.1161/CIRCULATIONAHA.116.027121

[8] E. A. Martin , A. Prasad , C. S. Rihal , L. O. Lerman , and A. Lerman , “Endothelial Function and Vascular Response to Mental Stress Are Impaired in Patients With Apical Ballooning Syndrome,” Journal of the American College of Cardiology 56, no. 22 (2010): 1840–1846. 21087714 10.1016/j.jacc.2010.03.107 PMC3786427

[9] I. Eitel , F. von Knobelsdorff‐Brenkenhoff , P. Bernhardt , et al., “Clinical Characteristics and Cardiovascular Magnetic Resonance Findings in Stress (Takotsubo) Cardiomyopathy,” Journal of the American Medical Association 306, no. 3 (2011): 277–286. 21771988 10.1001/jama.2011.992

[10] F. Rivero , J. Cuesta , M. García‐Guimaraes , et al., “Time‐Related Microcirculatory Dysfunction in Patients With Takotsubo Cardiomyopathy,” JAMA Cardiology 2, no. 6 (2017): 699. 28273298 10.1001/jamacardio.2016.5993 PMC5815019

[11] A. A. Vitin , L. Azamfirei , and D. Tomescu , “Perioperative Stress‐Induced (Takotsubo) Cardiomyopathy in Liver Transplant Recipients,” Journal of Critical Care Medicine 4, no. 2 (2018): 56–63. 30581996 10.2478/jccm-2018-0006 PMC6294992

[12] S. Agarwal , M. G. Bean , J. S. Hata , and M. R. Castresana , “Perioperative Takotsubo Cardiomyopathy: A Systematic Review of Published Cases,” Seminars in Cardiothoracic and Vascular Anesthesia 21, no. 4 (2017): 277–290. 29098955 10.1177/1089253217700511

